# Respiratory distress symptom intervention for non-pharmacological management of the lung cancer breathlessness–cough–fatigue symptom cluster: randomised controlled trial

**DOI:** 10.1136/spcare-2022-003924

**Published:** 2022-10-25

**Authors:** Janelle Yorke, Miriam J Johnson, Grant Punnett, Jaclyn Smith, Fiona Blackhall, Mari Lloyd Williams, Peter Mackereth, Jemma Haines, David Ryder, Ashma Krishan, Linda Davies, Aysha Khan, Alex Molassiotis

**Affiliations:** 1 Christie Patient Centred Research, The Christie NHS Foundation Trust, Manchester, UK; 2 Faculty of Health Sciences, The University of Manchester, Manchester, UK; 3 Wolfson Palliative Care Research Centre, University of Hull, Hull, UK; 4 Christie Patient Centred Research, Manchester University NHS Foundation Trust, Manchester, UK; 5 Primary Care, University of Liverpool, Liverpool, UK; 6 Nursing, Polytechnic University, Hong Kong, Hong Kong

**Keywords:** lung, dyspnoea, fatigue

## Abstract

**Objectives:**

In lung cancer, three prominent symptoms, such as breathlessness, cough and fatigue, are closely related with each other forming a ‘respiratory distress symptom cluster’. The aim of this study was to determine the clinical and cost-effectiveness of the respiratory distress symptom intervention (RDSI) for the management of this symptom cluster in people with lung cancer.

**Methods:**

A single blind, pragmatic, randomised controlled trial conducted in eight centres in England, UK. A total of 263 patients with lung cancer were randomised, including 132 who received RDSI and 131 who received standard care. To be eligible, participants self-reported adverse impact in daily life from at least two of the three symptoms, in any combination. Outcomes were change at 12 weeks for each symptom within the cluster, including Dyspnoea-12 (D-12), Manchester Cough in Lung Cancer (MCLC) and Functional Assessment of Chronic Illness-Fatigue.

**Results:**

At baseline, nearly 60% of participants reported all three symptoms. At trial completion the total trial attrition was 109 (41.4%). Compared with the control group, the RDSI group demonstrated a statistically significant improvement in D-12 (p=0.007) and MCLC (p<0.001). The minimal clinically important difference MCID) was achieved for each outcome: D-12 –4.13 (MCID >3), MCLC −5.49 (MCID >3) and FACIT-F 4.91 (MCID >4).

**Conclusion:**

RDSI is a clinically effective, low-risk intervention to support the management of the respiratory distress symptom cluster in lung cancer. However, the study did experience high attrition, which needs to be taken onto consideration when interpreting these results.

**Trial registration number:**

NCT03223805.

**WHAT IS ALREADY KNOWN ON THIS TOPIC**

- Patients with lung cancer experience symptoms that are described as distressing and impact quality of life. Breathlessness, cough and fatigue have been reported as a unique symptom cluster.

**WHAT THIS STUDY ADDS**

- We describe a randomised controlled trial of respiratory distress symptom intervention (RDSI) that demonstrated positive effects on self-reported breathlessness, cough and fatigue.

**HOW THIS STUDY MIGHT AFFECT RESEARCH, PRACTICE OR POLICY**

- We have shown that RDSI is an effective and low-risk intervention that could be implemented into practice. However, as with other palliative care studies we report high attrition; future work should explore how to minimise and manage this.

## Introduction

For patients with lung cancer, cure is rarely possible, and symptom control is a key part of management.^1^ Patients with lung cancer often experience multiple symptoms due to the disease and disease-related treatments or comorbidities. Common high burden symptoms include breathlessness, cough and fatigue, which have a deleterious effect on health-related quality of life.^2–4^

Three prominent symptoms—breathlessness, cough and fatigue—are closely related with each other forming a ‘respiratory distress symptom cluster’.^5^ One symptom will often interact adversely with one or both of the others, and occurrence of a symptom cluster appears to worsen patient outcomes.^6^ There are few oncology studies of prospective, controlled symptom-cluster intervention trials; lung cancer symptom management research largely focuses on a single symptom. Non-pharmacological breathlessness management techniques are clinically and cost-effective.^6–9^ Interventions such as exercise and acupressure are effective in the management of cancer-related fatigue,^10–12^ but these have not been robustly tested in lung cancer. Cough has received less attention as other cancer symptoms, which means that patients’ experience of this distressing symptom may not be optimally managed.^13 14^

We developed and feasibility-tested a multicomponent non-pharmacological intervention for the management of the breathlessness–cough–fatigue symptom cluster in lung cancer—respiratory distress symptom intervention (RDSI).^15–19^ We demonstrated that RDSI was acceptable to patients with lung cancer and indicated improved symptoms compared with usual care. We present a phase III randomised controlled trial (RCT) to assess the clinical and cost-effectiveness of RDSI. The primary hypothesis for this study was that, compared with participants who receive usual care, participants who receive the RDSI will report greater improvement in breathlessness, cough and fatigue at 12 weeks.

## Methods

### Trial design and participants

This was a multicentre pragmatic single-blinded, parallel group, RCT conducted in eight hospitals in England. Adult (18 or over) patients on any treatment or follow-up pathway who met the following criteria were invited to participate in the study: (1) diagnosis of intrathoracic malignancy (including small cell or non-small cell lung cancer, mesothelioma); (2) self-reported adverse impact in daily life from at least two of the three symptoms, in any combination; (3) if chronic obstructive pulmonary disease was present, it must have been stable; (4) WHO Performance Status 0–2 and (5) life expectancy greater than 3 months. Written informed consent was obtained prior to participation. Participants were identified through lung cancer multidisciplinary team meetings, lung cancer nurse specialists’ case lists and lung cancer outpatient clinics. Trial randomisation, monitoring and data management were conducted independently by the Manchester Clinical Trials Unit.

### Intervention

The development of the RDSI has been previously reported and tested for feasibility.^19^

The key intervention components included:

1. Controlled breathing techniques: consisting of diaphragmatic breathing exercises or pursed lip breathing, practised twice a day or used as needed beyond that for episodes of intense breathlessness and/or anxiety.
2. Cough suppression techniques: includes education (capacity for voluntary cough easing), identifying warning signs for cough and replacing with sips of water, modified swallow technique, huff cough technique or relaxed throat breathing.
3. Acupressure: a small number of acupressure points were taught: L7, L9 (for cough and dyspnoea, located on the wrist areas), LI4 (for energy, located in hand), CV21 and CV22 (for cough and dyspnoea, located in sternum) and ST36 (for energy, located in the knee). Patients could select any of these points in any combination to apply pressure for 1 min at least twice a day for symptom relief.
4. Exercise: Individually tailored exercise plan, for example, walking incrementally increasing distances in their local environment, and incorporating breathing techniques as required.

Healthcare professionals, including nurses, physiotherapists and occupational therapists, were trained in the delivery of the RDSI as part of their usual clinical practice with patients with lung cancer. The training, an intervention protocol, specific trainer manual and video (Digital Versatile Disc) of the techniques were provided by the research team. RDSI trained staff at each site were able to train other healthcare professionals who were part of the lung cancer team to deliver the intervention. This pragmatic approach differed from the feasibility trial, where clinicians were specifically employed as research assistants to deliver the intervention.

### Randomisation

Following consent participants were allocated to a trial arm through computer-generated randomisation with a 1:1 allocation ratio and a random element controlling for three variables: (1) treatment centre; (2) disease (mesothelioma/primary/secondary lung cancer) and (3) treatment intent (radical vs palliative). Allocations were made to the arm that would reduce imbalance, with probability of 0.75 (0.5 when imbalance scores were tied).

### Outcomes

The three coprimary outcomes were change at 12 weeks for each symptom within the cluster, assessed using separate breathlessness, cough and fatigue validated questionnaires, in the RDSI group compared with the standard care group.

Breathlessness perception: Dyspnoea-12 (D-12) was the primary outcome for breathlessness.^20^ The D-12 assesses total breathlessness severity, including its physical discomfort and emotional consequences. Total scores range from 0 to 36, with higher scores indicating more severe breathlessness. It is validated for use in lung cancer^21^), with a minimal clinically important difference (MCID) of 3 points.

Cough perception: The Manchester Cough in Lung Cancer (MCLC) scale was the primary outcome for cough. It consists of 10 items ranging from 0 to 40, with higher scores indicating more severe cough.^22^ It has a reported MCID of 3 points.

Fatigue perception: The Functional Assessment of Chronic Illness-Fatigu was the primary outcome for fatigue.^23^ This 13-item scale has scores ranging from 0 to 52, and with lower scores indicating more severe fatigue. It has a reported MCID of four points.

Secondary outcome measures consisted of the following:

Coping with Symptoms: A Numerical Rating Scale scored 0–10, where 0 indicated ‘not coping at all’ and 10 indicated ‘completely coping’ with the given symptom, which was completed separately for breathlessness, cough and fatigue. It was developed for use in this study.

Hospital Anxiety and Depression Scale (HADS): A 14-item scale assessing anxiety with seven items and depression with a further seven items. Each item is answered on a 4-point scale (0–3). Scores on each subscale thus range between 0 (no symptoms) and 21 (numerous and severe symptoms). Higher scores are indicative of more anxiety/depression.

Euro-Qual 5-level version (EQ5D): A standardised instrument for use as a measure of health outcome.^24^ The EQ5D was used to assess a preference-based measure of health-related quality of life, which enabled the calculation of quality of life-years for use in the cost-effectiveness analysis.

Healthcare utilisation: Assessment of resource use was assessed via patient recall using a standardised instrument at baseline (previous 4 weeks), week 4 and week 12 postrandomisation. Utilisation of resources measured included: planned hospital or hospital overnight stays, hospital emergency visits and admissions (per night), GP and other community service visits.

### Sample size

The target sample size calculation informed by our feasibility data was 258 patients (129 per arm). This provides 80% power, with a one-tail 0.05 overall significance level (0.0167 per symptom), to detect improvements in MCIDs in any of the three clustered symptoms assuming (MCID=3, SD=9.0, r=0.60) for breathlessness, (MCID=3, SD=8.5, r=0.55) for cough and (MCID=4, SD=11.7, r=0.60) for fatigue, respectively. A 20% attrition rate was included in the calculation.

### Statistical methods

Descriptive statistics and frequency distributions were calculated for the participants’ demographic and clinical characteristics. Hypotheses were tested on an intention-to-treat basis. The effect of the three coprimary outcome measures at week 12 were assessed using analysis of covariance models for each component with focus on the trial arm effect after adjustment for baseline questionnaire values and two factors controlled for in the randomisation algorithm; treatment centre and disease (primary and secondary lung cancer were merged as there was a single secondary case). The third factor ‘treatment intent’ was not adjusted for, as it turned out to be a somewhat confusing mixture of historical and prospective intents. The coprimary effect estimates were differences in adjusted means, reported with a 96.7% CI and a two-tailed threshold for significance of p=0.033 (=2*0.05/3). The power calculations in the protocol were based on one-sided tests and an overall 0.05 significance level, however, two-sided tests and CIs are used throughout this report. Secondary outcome analyses repeated the primary analysis method for all outcomes. The effect estimate was the difference in adjusted means, reported with a 95% CI and a two-tailed threshold for significance (p=0.05).

Multiple imputation was used as a principled approach for analysis. One hundred imputation sets were generated and used for all the clinical outcome analyses but participants known to have died prior to week 12 were deliberately excluded. The variables used in the imputation algorithm included all questionnaire scores at baseline, week 4 and week 12, age, gender, treatment centre and disease. Participants dying before week 12 were removed, as we did not wish to impute counterfactual values. The effect of missing values was assessed by comparing the numbers and percentages of patients with missing values in the two arms of the trial. Logistic regression was used to assess potential factors affecting drop-out.

The economic analysis was conducted from the National Health Service (NHS) (costs) and patient’s (health benefit) perspective over the 12 weeks of scheduled follow-up. The analysis used individual patient level cost and health benefit Quality Adjusted Life Years (QALY) data from all participants recruited and starting allocated treatment in the trial. QALYs were estimated from the five-level version of the EQ-5D-5L and associated utility tariffs^24 25^ completed at baseline, 4 and 12 weeks. Given the nature of the RDSI intervention, it was assumed additional service use to provide this was captured in the participant service use questionnaire. The costs of NHS and social care services were derived from published national unit cost data for 2018-19.^26^ Descriptive comparisons of costs and QALYs between treatment arms at the patient level are reported, but not tested for statistically significant differences. Regression analysis for the cost analysis included prior costs and that for QALYs included the baseline EQ-5D thermometer score. The primary analysis used participants with complete cost and QALY data. The estimates of incremental costs and outcomes from the regression were bootstrapped to simulate 10 000 pairs of net cost and net outcomes of the RDSI group. The bootstrapped data were used for a cost-effectiveness acceptability analysis, to estimate the probability that RDSI is cost-effective compared with usual care. The willingness-to-pay threshold to gain 1 QALY was £15 000 for the primary analysis, with a range of £0–£30 000 per QALY gained. This reflects a lack of consensus about the threshold willingness to pay value for the UK.^26^

## Results

**Figure 1:**
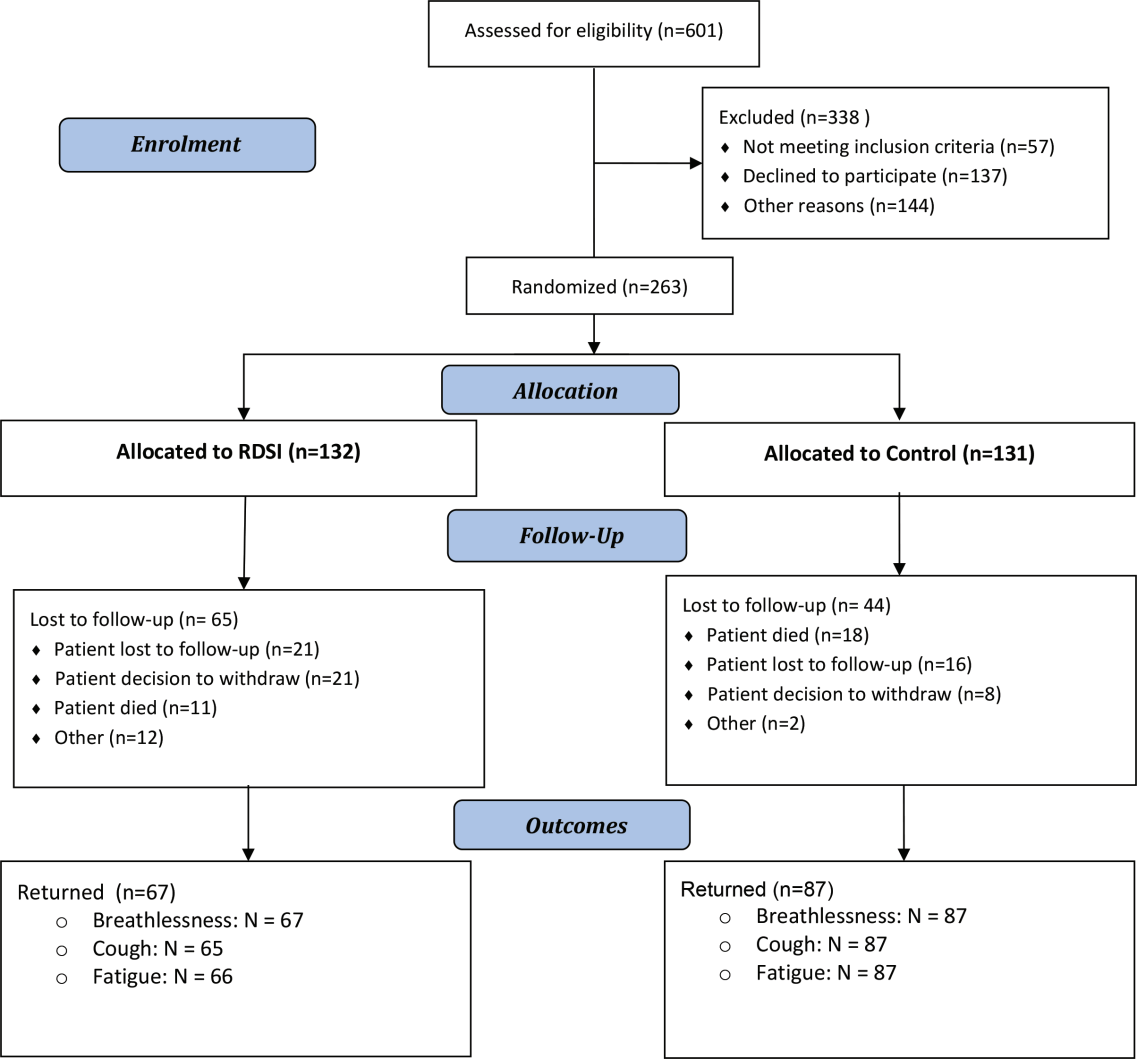
Trial Consort diagram.

Of 601 patients with lung cancer were assessed for eligibility, 263 were randomised (RDSI group=132 and control group=131) (figure 1). Most participants had a primary diagnosis of NSCLC (230; 87.5%), had a WHO status 1 (150, 57%) and were treated with palliative intent (159, 60.5%) (table 1). The mean age of the participants was 69 and approximately 50% were female (table 1). At trial completion (12 weeks), 67 (50.8%) RDSI group participants and 87 (66.4%) control group participants remained; the total trial attrition was 109 (41.4%) and was higher in the RDSI group. A total of 29 (11%) patients died during the trial (RDSI: 11 (8.3%); control group: 18 (13.7%)). Attrition due to death was less in the RDSI arm, but ‘lost to follow-up’ or ‘patient decision to withdraw’ was more common. There was a notable difference in attrition rates across the 8 participating sites: one site recruited 15 patients and 73% did not complete, and another site recruited 13 patients and 54% did not complete.

**Table 1:** Demographics and clinical characteristics of patients in the two study groups

	RDSI intervention		Control arm		All participants	
	N	Mean (SD) or frequency, %	N	Mean (SD) or frequency, %	N	Mean (SD) or frequency, %
Female	67	50.8%	65	49.6%	132	50.2%
Male	65	49.2%	66	50.4%	131	49.8%
Age	132	69.2 (8.70)	131	69.5 (10.20)		
Smoking						
Never smoked	18	13.6%	16	12.2%	34	12.9%
Ex-smoker	100	75.7%	96	73.3%	196	74.5%
Smoker	14	10.6%	19	14.5%	33	12.5%
COPD						
Yes	41	31.1%	43	32.8%	84	31.9%
No	91	68.9%	88	67.2%	179	68.1%
WHO PS						
0	20	15.15%	21	16.03%	41	15.59%
1	79	59.85%	71	54.20%	150	57.03%
2	33	25.00%	39	29.77%	72	27.38%
Diagnosis						
Mesothelioma	17	12.88%	15	11.45%	32	12.17%
Primary LC	115	87.12%	115	87.79	230	87.45%
Secondary LC	0	0%	1	0.76%	1	0.38%
Time since diagnosis (years)*	131	1.8 (1.8)	130	1.8 (1.9)		
Current Opiates						
Yes	42	31.8%	41	31.3%	83	31.6%
No	90	68.2%	90	68.7%	180	68.4%
Current benzodiazepines						
Yes	12	9.1%	10	7.6%	22	8.4%
No	120	90.9%	121	92.4%	241	91.6%
Completed treatment intent						
Palliative	79	59.8%	80	61.1%	159	60.5%
Radical	53	40.2%	51	38.9%	104	39.5%

*Two patients did not have the date of diagnosis recorded, so data only available from 224 patients.

COPD, chronic obstructive pulmonary disease; LC, Lung Cancer; PS, Performance Status; RDSI, respiratory distress symptom intervention.

Almost 60% of participants reported all three symptoms at baseline (table 2).

**Table 2:** Self-reported symptoms at baseline

Symptoms at baseline	No	%
Breathlessness	257	97.7
Cough	166	63.1
Fatigue	259	98.5
All three symptoms	156	59.3

Table 3 shows the coprimary and secondary outcomes by trial arm at baseline, week 4 and week 12.

**Table 3:** Coprimary and secondary outcomes by trial arm

Outcome	Mean (SE)		Adjusted trial arm effect		
	RDSI	Control	β	95% CI	P value*
Breathlessness (Dyspnoea-12)					
Baseline	16.71 (0.93)	15.31 (0.91)			
4 weeks	12.86 (1.20)	14.95 (1.14)	−3.15	−5.82 to −0.48	0.021
12 weeks	11.45 (1.31)	14.35 (1.14)	−4.13	−7.09 to −1.18	0.007
Cough (MCLC)					
Baseline	13.69 (0.88)	13.95 (0.92)			
4 weeks	11.10 (1.09)	15.28 (1.04)	−4.02	−6.37 to −1.68	0.001
12 weeks	8.47 (1.29)	14.04 (1.08)	−5.49	−8.43 to −2.55	<0.001
Fatigue (FACIT-F)					
Baseline	24.32 (0.99)	24.07 (1.07)			
4 weeks	27.42 (1.69)	23.90 (1.36)	3.47	−0.38 to 7.31	0.077
12 weeks	28.62 (2.09)	23.72 (1.44)	4.91	0.24 to 9.57	0.039
NRS coping with symptoms (breathlessness)					
Baseline	6.29 (0.23)	6.09 (0.20)			
4 weeks	6.67 (0.34)	5.77 (0.30)	0.81	−0.03 to 1.65	0.424
12 weeks	6.97 (0.39)	5.85 (0.35)	1.07	0.13 to 2.01	0.026
NRS coping with symptoms (cough)					
Baseline	6.43 (0.26)	6.36 (0.25)			
4 weeks	7.12 (0.41)	6.29 (0.34)	0.78	−0.21 to 1.77	0.120
12 weeks	7.41 (0.45)	6.28 (0.39)	1.18	0.07 to 2.30	0.037
NRS coping with symptoms (fatigue)					
Baseline	4.97 (0.24)	5.29 (0.24)			
4 weeks	5.96 (0.41)	5.17 (0.29)	0.96	0.03 to 1.89	0.042
12 weeks	5.78 (0.48)	5.25 (0.37)	0.62	−0.52 to 1.76	0.284
HADS Questionnaire: Anxiety					
Baseline	7.33 (0.42)	6.81 (0.43)			
4 weeks	6.81 (0.60)	7.73 (0.46)	−1.32	−2.53 to −0.12	0.031
12 weeks	6.40 (0.57)	7.19 (0.52)	−1.20	−2.45 to 0.05	0.061
HADS Questionnaire: Depression					
Baseline	6.91 (0.34)	7.16 (0.36)			
4 weeks	7.13 (0.51)	7.69 (0.44)	−0.40	−1.54 to 0.73	0.482
12 weeks	6.90 (0.73)	7.63 (0.53)	−0.60	−2.28 to 1.09	0.482

Unadjusted means and standard errors for coprimary and secondary outcomes by trial arm and time point derived from 100 imputation sets. Adjusted trial arm effects from ANCOVA models are also provided at 4 and 12 weeks, respectively.

*Reference two-tail significance levels are (2*0.05/3) = 0.033 for the 3 coprimary outcomes (Breathlessness, Cough and Fatigue) and 0.05 for secondary outcomes.

ANCOVA, analysis of covariance; FACIT-F, Functional Assessment of Chronic Illness-Fatigue; HADS, Hospital Anxiety and Depression Scale; MCLC, Manchester Cough in Lung Cancer; NRS, Numerical Rating Scale; RDSI, respiratory distress symptom intervention.

### Changes in breathlessness, cough and fatigue severity ratings between the two groups at 12 weeks

Breathlessness: The adjusted mean difference in D-12 scores between the RDSI and control was −4.13 (96.7% CI −7.35 to −0.91; p=0.007), indicating evidence of a clinically (>3) and statistically significant benefit (p<0.033) in the RDSI arm.

Cough: The adjusted mean difference in MCLC scores between the RDSI and control was −5.49 (96.7% CI −8.70 to −2.29; p<0.001), indicating evidence of a clinically (>3) and statistically significant benefit (p<0.033) in the RDSI arm.

Fatigue: The adjusted mean difference in MCLC scores between the RDSI and control 4.91 (96.7% CI −0.18 to 9.99; p=0.039), indicating evidence of a clinically (>4) and statistically significant benefit (p<0.033) in the RDSI arm, although the lower CI was close to zero and the p value was close to the significance level.

**Figure 2:**
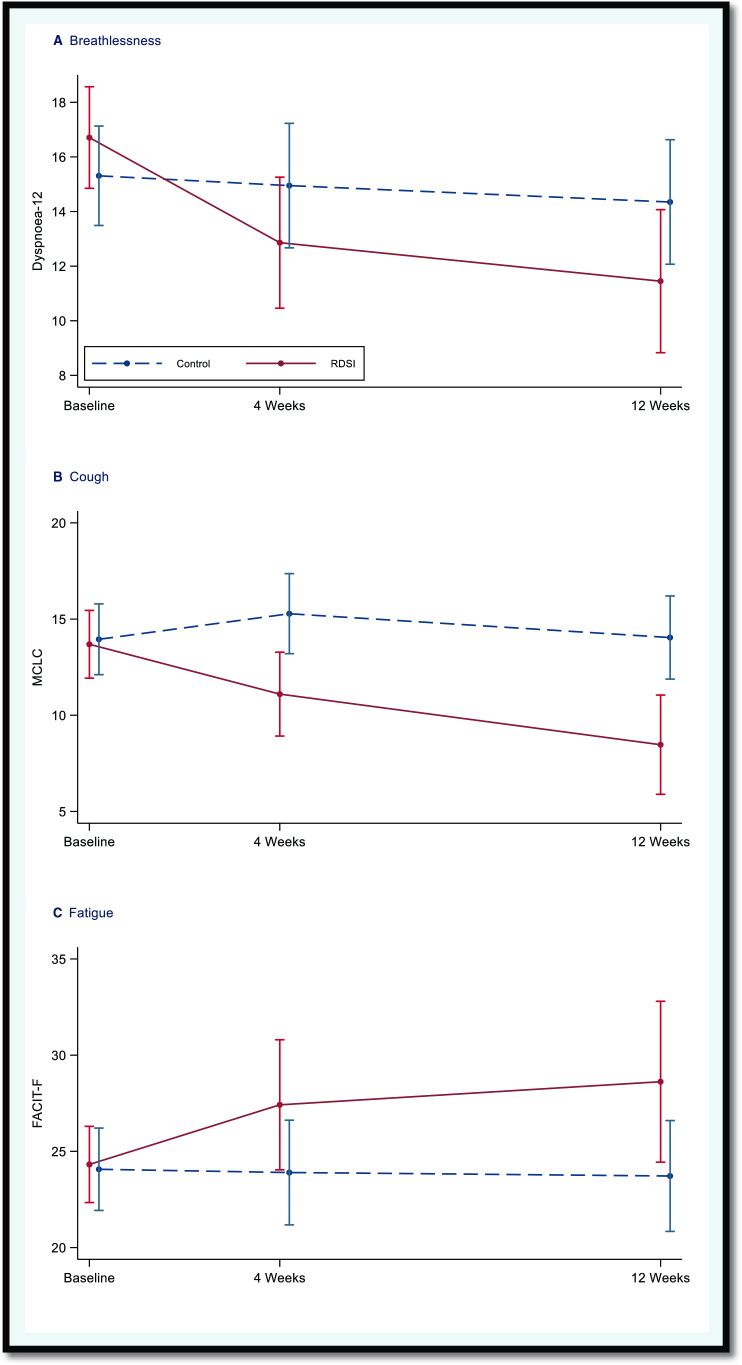
Changes over the study period for the coprimary outcomes. Unadjusted means and approximate 95% CIs (mean±2*SE) for the coprimary outcomes by trial arm and time point derived from 100 imputation sets. FACT-F, Functional Assessment of Chronic Illness-Fatigue; MCLC, Manchester Cough in Lung Cancer; RDSI, respiratory distress symptom intervention.

See table 2 and figure 2 for a summary of the changes in outcome assessments across the study period.

### Change in self-reports of coping with the cluster symptoms between the two groups at 12 weeks

Participants reported a clinically (>1) and statistically significant improvement for coping with breathlessness (mean difference 1.07, p=0.026), and cough (mean difference 1.18, p=0.037), but not for fatigue (mean difference 0.62, p=0.284).

### Change in anxiety and depression between the two groups at 12 weeks

The mean adjusted scores from the anxiety subscale for the HADS questionnaire for the RDSI arm was 6.21 compared with 7.40 in control. The adjusted mean difference was −1.19 (95% CI −2.45 to 0.05; p=0.061), indicating no statistically significant difference. The mean adjusted scores from the depression subscale for the HADS questionnaire for the RDSI arm was 6.96 compared with 7.56. The adjusted mean difference was −0.60 (95% CI −2.28 to 1.09; p=0.482), indicating no statistically significant difference.

### Cost-effectiveness

Overall, 257 participants completed one or more items on the EQ-5D and 255 completed one or more items of the service use questionnaire at baseline. Of the 154 participants who completed follow-up 102 had complete cost and QALY for the 12-week follow-up (61/87 (70%) control arm; 41/67 (61%) RDSI arm). Table 4 reports the descriptive analysis for the 102 participants with complete data as well as the bootstrapped data for the primary and sensitivity analyses. The cost-effectiveness acceptability analysis indicated that the probability RDSI is cost-effective was 14% if decision makers are willing to pay £15 000 to gain 1 QALY (range 6%–23%). The sensitivity analysis including indicators of incomplete cost/QALY data (for those participants who completed follow-up) indicated similar results.

**Table 4:** Mean QALYs and costs and cost-effectiveness acceptability analysis, UK £’s, 2018–2019 prices

Follow-up	Control (61/132, 46%)				RDSI (41/131, 31%)			
	Mean	SE	95% CI		Mean	SE	95% CI	
QALYS								
Baseline to 4-week follow-up	0.044	0.002	0.040	0.047	0.052	0.003	0.046	0.057
4–12 weeks follow-up	0.088	0.004	0.080	0.095	0.104	0.005	0.093	0.114
Baseline to 12 weeks follow-up	0.133	0.005	0.122	0.143	0.154	0.007	0.140	0.169
**Costs**								
Baseline to 4-week follow-up	£461	£134	£195	£726	£784	£522	£<1	£1820
4–12 weeks follow-up	£659	£176	£310	£1008	£1450	£812	£<1	£3060
Baseline to 12 weeks follow-up	£1120	£242	£639	£1600	£2234	£1320	£<1	£4851
**Bootstrapped estimates**	**Net QALY or Cost of RDSI vs control**		**95% percentiles**		**Probability RDSI is cost-effective**			
					**WTPT=** **£0 per QALY gained**	**WTPT=** **£15 000 per QALY gained**	**WTPT=** **£30 000 per QALY gained**	
Primary analysis (complete cost and QALY data, n=102)								
Net QALYs	0.019		0.001; 0.037		0.06	0.14	0.23	
Net cost	£1401		−£544; £3347					
Sensitivity analysis (available cost and QALY data, indicator for incomplete data, n=147)								
Net QALYs	0.015		0.001; 0.030		0.06	0.13	0.21	
Net cost	£1142		-£824; £3108					

QALY, Quality Adjusted Life Years; RDSI, respiratory distress symptom intervention; WTPT, willingness-to-pay threshold.

## Discussion

This is one of the few RCTs to report effectiveness of a multicomponent intervention that targets a symptom cluster in lung cancer. We showed that the RDSI is a clinically effective, low-risk intervention to support the management of the respiratory distress symptom cluster in lung cancer. The main effects were significant for breathlessness and cough; further work is needed to ascertain the potential to further benefit the experience of fatigue.

There was a higher-than-expected drop-out rate, which was also larger in the RDSI group. The total attrition for the RDSI in the feasibility study was 29.7% at 12 weeks,^19^ compared with 41.4% for the trial. Reasons for this difference are not entirely clear although there were changes to the trial protocol between studies, which may have contributed to this. First, the face-to-face teaching sessions were decreased from two to one (second session replaced with a telephone call). Recent evidence suggests that palliative care patients prefer, and benefit from, symptom interventions that consist of one training session compared with three; likely due to minimisation of intervention and trial burden.^27^ However, the latter study targeted breathlessness as a single symptom as opposed to a more complex multicomponent intervention that targeted a symptom cluster. The trade-off between trial burden and building participant confidence to master a complex intervention may have impacted trial attrition; in that our participants required more intense and frequent training sessions to fully grasp the intervention. This raises an important implication when developing multimodal interventions that target a cluster of symptoms.

Participants in the feasibility trial reported high compliance with the RDSI components, although following two face-to-face sessions.^19^ We did not assess intervention fidelity in this main trial. There were no features in between-group characteristics at week 12 to indicate harm from the RDSI. Future study should explore reasons for study attrition in more detail.^28^ The attrition rate observed within this study, however, is comparable to other supportive and palliative care trials.^29^

A second key difference between the feasibility and main trial was the training of healthcare professionals to deliver the intervention as part of their day-to-day patient care. For the feasibility study, healthcare professionals were employed as researchers on the trial—they were trained to deliver the intervention to patients without the potential limitation of implementing the RDSI into busy clinical schedules. Our pragmatic approach was important to assess the effectiveness of the RDSI in a ‘real life’ scenario.

This study consisted of three coprimary outcomes; one for each symptom within the cluster. We chose the D-12, MCLC scale and FACIT-F based on our previous feasibility study and evidence of validity for each instrument in the lung cancer patient population. Some symptom cluster researchers have advocated the use of composite scores^30 31^; however, this may not fully capture changes within each symptom that characterise the change in the total score. In the absence of a more robust way to measure symptom clusters, we chose to look at each symptom separately, adjusting the p value accordingly.

The D-12 scores showed a medium effect size for the RDSI exceeding the MCID of 3 units. The RDSI included well-established self-management techniques for breathlessness including breathing exercises and relaxation, physical activity and certain acupressure points.^30 32 33^ There is a growing body of work that describes breathlessness targeted interventions that are multicomponent and delivered at a service level with a multidisciplinary team approach; referred as ‘holistic services’.^34^ Such studies also include single breathlessness interventions not dissimilar to the RDSI.

A medium effect size was also observed for cough easing; our previous feasibility study showed that the MCID was 3 units; which was exceeded. At the time of finalising the RDSI for this phase III trial, the evidence base for non-pharmacological cough easing techniques was limited.^35^ Further studies based on speech and language therapy confirm the benefit of such techniques.^36^ However, it is important to note that these studies included patients with a confirmed diagnosis of refractory chronic cough, and the intervention was delivered by skilled therapists. In this pragmatic trial, we applied a high-level approach to the cough easing techniques that could be taught to patients by non-specialists for self-management. The observed benefits with RDSI provide further evidence that even simple exercises can lead to significant improvements in the cough experience.

Cancer-related fatigue is described as a persistent sense of physical and emotional tiredness or exhaustion and is not directly linked to recent activity nor is it relieved by rest^37^; making it a difficult symptom to manage. However, there is good evidence of the benefit for the different components of the RDSI that targeted fatigue. Although the predetermined MCID of 4 units for the FACIT-F^38^ was achieved (4.91), a statistically significant effect was not observed when Bonferroni correction applied. The activity/exercise component of the RDSI did not include a specific exercise programme, and it may be that a more structured approach with regular follow-up is required.

The term ‘coping’ was used based on feedback from patients during the feasibility trial as it was said to describe a patient’s overall perception of symptom management. We found that patients in the RDSI group reported a greater sense of ‘coping’ with breathlessness and cough, but not for fatigue compared with the control group. Henoch *et al* identified the importance of ‘coping capacity’ in helping patients with lung cancer manage the palliative phase of disease^39^; the RDSI may play an important part in increasing this.

In contrast to the positive effect of the RDSI on the symptom-cluster severity and ability to cope, there were no significant changes shown for psychological distress assessed with HADS; although anxiety had a non-significant trend in improvement. Other trials aimed at improving a single symptom such as breathlessness or respiratory-type symptom clusters have shown mixed results for impact on general anxiety and depression.^9 40^ The interventions used in our study target specific symptoms, and it may be that additional psychological support, such as cognitive–behavioural therapy, is needed to further benefit people demonstrating anxiety and depression. We recommend screening for psychological distress with the HADS at trial entry and providing additional input as required.

Overall, the economic analysis suggests that RDSI is not cost-effective. However, the net costs in particular indicate a high level of variation. Combined with the relatively small sample with complete cost and QALY data and the limitations of the trial this suggests that the results of the economic analysis are uncertain rather than conclusive.

### Limitations

This pragmatic RCT has a number of limitations. For this trial, the intervention was delivered by clinicians in real-time clinical practice. This approach was important because we wanted to assess the impact of the RDSI when delivered as part of busy clinical schedules; however, this did result in some delay to each part of the intervention being delivered on time. Due to the type of intervention, blinding patients to randomisation group was not possible; however partial blinding was adhered to for outcome assessors and the analysis.

The trial had high attrition that was higher in the RDSI group. This may suggest that the multicomponent intervention was complex for some patients to sustain for a long time, or that those dropping out may not have observed a perceived benefit. The differential attrition between sites may also reflect differences in necessary support for participants to fully engage with trial procedures and interventions. Intervention compliance from the participants perspective was not monitored in the current study so we are not able to correlate the RDSI drop-out with intervention fidelity. As the attrition rate is at the level which may introduce bias and resulted in loss of power^41^ (which may have influenced the non-statistically significant findings for fatigue) considerations as to how best minimise and handle missing data in future work are important.

## Conclusion

This study provides new and important evidence for the management of a common and distressing symptom cluster experienced by patients with lung cancer. Breathlessness and cough were the two symptoms improving significantly and these may stronger characterise the respiratory distress clusters, and it may be better to focus future work on these highly related symptoms rather than adding other related symptoms too. The incorporation of the intervention in clinical routine shows that its components can be used as standard care and provide symptom benefit to patients in a low-cost self-management approach that is of low risk too.

## Data Availability

Data are available on reasonable request.
